# Insularity, character traits and local political interference: the pivotal players of the Sardinia regional health service’s progressive decline

**DOI:** 10.3389/fpubh.2026.1864788

**Published:** 2026-08-18

**Authors:** Armando Bartolazzi

**Affiliations:** Department of Pathology and Molecular Medicine, St. Andrea University Hospital, Rome, Italy

**Keywords:** evidence-based health policies, hospital network model, hub & spoke, insularity syndrome, local health authorities, regional health policy

## Abstract

Sardinia is a peculiar Italian autonomous region characterized by a small population, a unique genetic heritage and challenges related to socioeconomic isolation due to insularity. Despite the significant financial resources allocated annually for the Regional Health Service its performance is slowly getting worse. As autonomous insular region, Sardegna represents an ideal “experimental laboratory” for identify the major determinants of this failure. The character traits of Sardinian people, strongly influenced by centuries of colonization and socio-political adaptation, the challenges of insularity, and the health policies adopted at regional and local level, collectively contribute to the progressive failure of the Regional Health Service. The aim of this contribution is to analyze the effects of these factors on the Regional Health Service’s performance and to identify corrective actions to reverse the negative trend.

## 1 Introduction

The Italian region Sardinia covers 24.100 square kilometers and is the second-largest island in the Mediterranean Sea, after Sicily. The population of 1.562.380 inhabitants is mostly concentrated in the metropolitan areas of Cagliari and Sassari; 28 % is over 65, with several blue zones of great scientific interest for the presence of healthy centenarians (1).

The population density is approximately 65 inhabitants per square kilometer.

With the Special Statute of the Autonomous Region of Sardinia, approved by constitutional law no. 3 of February 26th, 1948, the island became an autonomous region, acquiring a broad degree of autonomy in matters of local administration, education, culture, economy, and healthcare.

The region, therefore, directly provides funding for the Regional Health Service (RHS), which is universal and public. The financial resources allocated annually by the Regional Government to guarantee public healthcare and social services, amount to approximately four4 billion € /year (€11.6 billion have been allocated for three-year period 2026–2028) and derive primarily from taxation imposed on the population.

As reported by the Annual National Outcome Plane (NOP), published each year by the National Agency for Healthcare (AgeNaS), a government administrative body operating under the Italian Ministry of Health, despite significant economic resources available so far to guarantee health services, Sardinia is ranked among the last in the national ranking of the Regions, when the ability to provide high-quality Essential Packages of Care (EPC) is considered (2). These data are also confirmed by a highly qualified independent agency called GIMBE (the Italian Group for Evidence-Based Medicine) in the 8th GIMBE Report on the National Health Service published in October 2025 (3).

**Figure 1:**
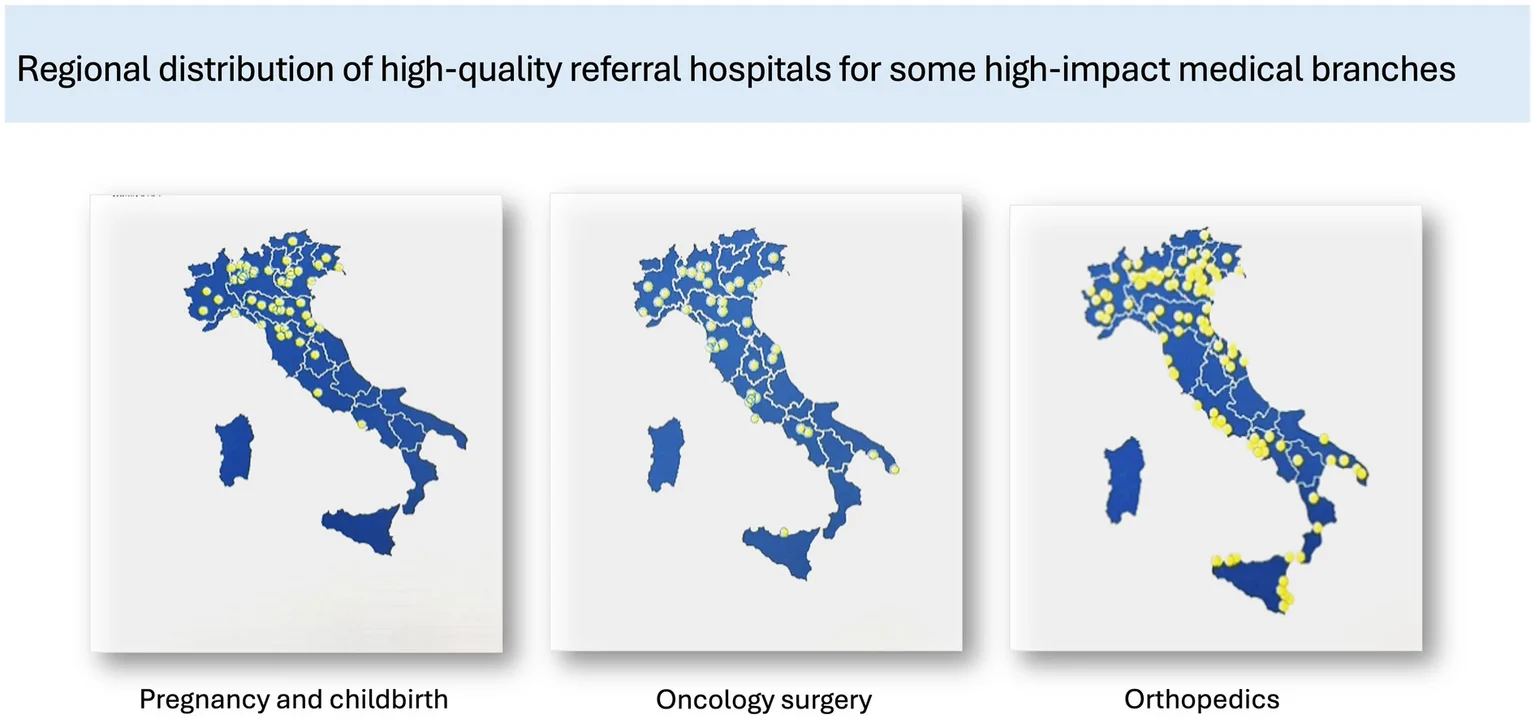
Regional distribution of high-quality referral hospitals for some high-impact medical branches. No referral hospitals are detected in Sardinia in the cardio-vascular sector also (data not shown). Only a reference center for neurology has been indicated in the North-West territory (data not shown). From the Annual Report 2025 (NOP) of the National Agency for Health Services (AgeNaS), Italian Ministry of Health [modified; https://www.agenas.gov.it and https://pne.agenas.it/home; Ref. (2)].

EPC establish the types of services that each RHS must provide to all citizens, either free of charge or for a small fee (4). Currently, access to the EPC varies significantly across the Italian regions. Waiting lists, organizational differences, and structural and technological shortcomings make the right to healthcare less certain for those living in some regions of the Country, with Sardinia being among the most critical one (2, 3). The National Outcomes Plan (NOP) edited by AgeNaS, evaluates the quality, effectiveness, and equity of regional hospital care based on hundreds of indicators. Table 1 shows the Sardinia rankings among the 21 Italian regions during the period from 2005 to 2025 according to the respective NOP’s data. Figure 1 shows some of the major criticalities with high impact on the population’s health, compared to other Italian Regions derived by the NOP 2025. Looking at the 2005–2025 trend, Sardinia has consistently remained in the lower half of the rankings, with a deterioration in certain indicators due to structural problems:

1. Low procedure volumes across most regional hospitals, resulting in fragmented caseloads.
2. Insular geography and the resulting outflow of patients to other regions for care (approximately 8% for highly complex procedures).
3. Critical timeframes for interventions regarding femur fractures and cardiac surgery.

**Table 1:** Sardinia’s ranking in the NOP (2005–2025): overall assessment.

Year	Ranking (among 21 regions)	NOP summary assessment	Major notes
2005	14	Medium	NOP just activated, mostly quantitative data; limited outcomes data. Criticalities regarding 30-day mortality for AMI (myocardial infarction) and treatment times for femoral fractures
2010	16	Medium-low	Low surgical oncology volumes High passive patient outflow to Lazio and Lombardy
2015	17	Medium-low	Issues regarding timeliness of femoral surgery, cesarean sections, and heart failure mortality.
2020	18	Low	Strong COVID impact All indicators are worsening
2025	17–19 (data 2023–2024)	Medium-low	Slight post-COVID recovery, but critical issues in high-impact sectors: Cardiovascular area 30-day mortality rate for AMI (Sardinia: 9.8%) (Italian average: 8.3%) Musculoskeletal care Femoral fracture treatment within 2 days: (Sardinia 52%) (Italian average 68%) Perinatal care Cesarean sections: (Sardinia 32.1%) (Italian average 21.7%) Oncological surgery volumes: Breast, colon, and lung surgical procedures below the minimum threshold in the majority of hospitals

https://www.salviamo-ssn.it/attivita/rapporto/8-rapporto-gimbe [ref. (3)].

Regarding gender-specific medicine institutionalized through framework Law 3/2018, proposed by the Italian Ministry of Health and followed by significant policy implementation measures at National level, such as the establishment of the three-year National Plan for Gender-Specific Medicine (2021–2023) and the National Observatory on Gender-Specific Medicine at the Italian National Institute of Health, various Local Health Authorities (ASLs) in Sardinia are introducing gender-sensitive outpatient clinics and care pathways, particularly in the fields of oncology, cardiology, and rare diseases. To date, the only available regional data on the impact of these policies can be derived from the AgeNaS NOP 2024. (Since 2019, the Agency has published hospital outcomes disaggregated by gender, across various indicators).

NOP 2024 reveals persistent gender inequalities disadvantaging women in several indicators (i.e., AMI and quick treatment of femoral fractures). While the 2015–2023 trend shows slight improvement, the region ranks 17th–19th on gender-specific indicators in 2024 (unaggregated data). The widest gap concerns PTCA (Percutaneous transluminal coronary angioplasty) procedures for women with STEMI [ST-segment elevation myocardial infarction; (2)].

Specific investments and implementation of evidence-based health policies for Gender Medicine are essential to improve this trend as well.

**Figure 2:**
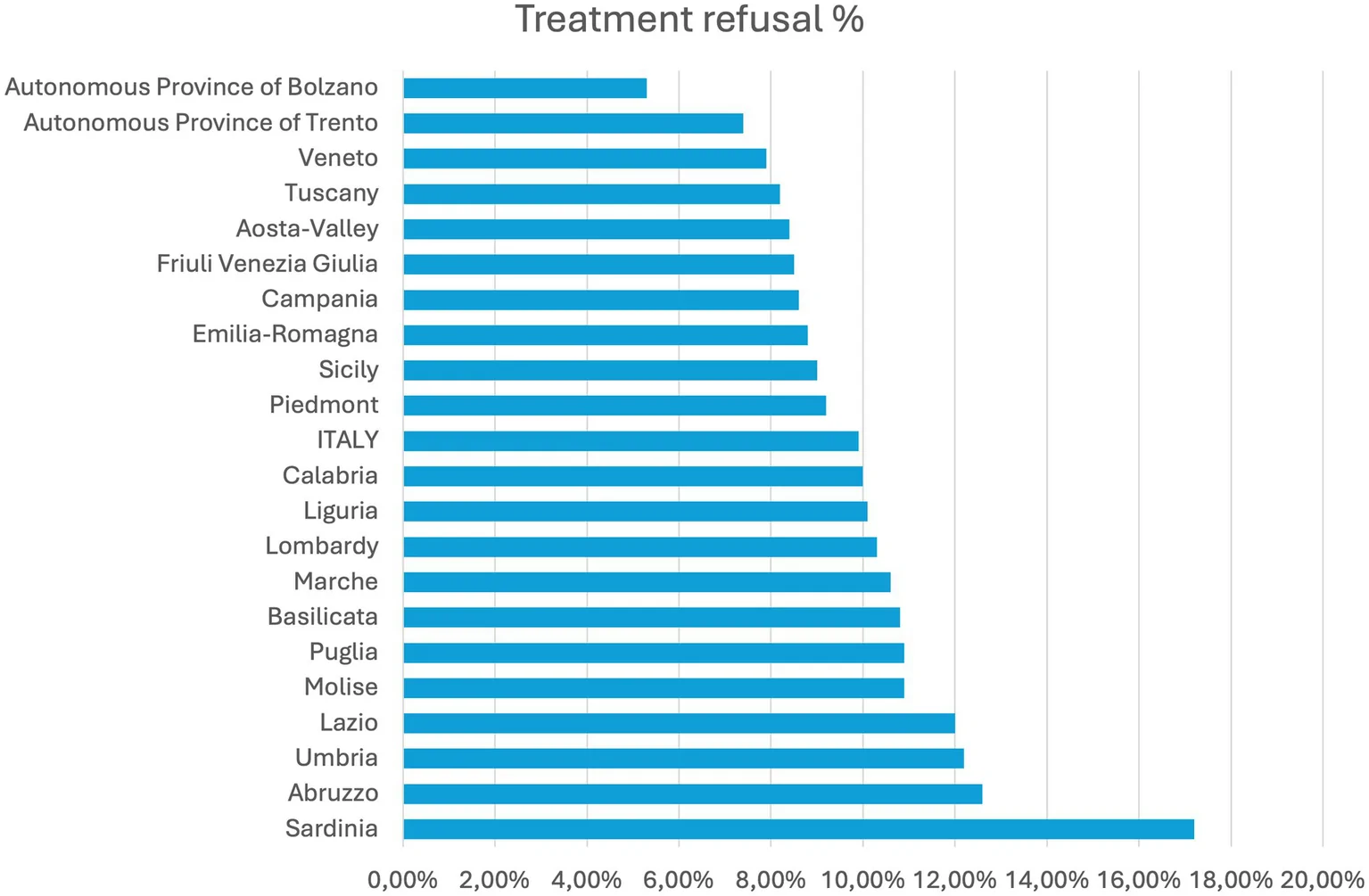
Refusal of healthcare by the Italian population in different regions (8th GIMBE Report on the National Health Service, year 2024—GIMBE: The Italian Group for Evidence-Based Medicine [modified; https://www.salviamo-ssn.it/attivita/rapporto/8-rapporto-gimbe; Ref. (3)].

Sardinian citizens often turn outside the region for their healthcare needs, because unable to find adequate solutions in house. Confirming this, the region spent about €100 million in the year 2024 on extra-regional healthcare mobility, while 17% of the Sardinian population gives up on treatment (5) (Figure 2).

As an island with an autonomous Regional Government, Sardinia is well-suited to a detailed analysis of the social and political dynamics that despite the significant financial resources allocated yearly, have prevented for too long the provision of good healthcare services to the population.

The present analysis derives from a direct political experience on-site as Regional Councilor for Health and Social Policies from April 2024 to December 2025 and takes into consideration the insularity related problems, the healthcare policies adopted at regional and local levels and some peculiar character traits of the Sardinian people, in particular the so called “colonial reticence” induced by centuries of colonization and exploitation of resources, that makes peer comparison across borders difficult. All these factors play as key determinants of the poor performance of the Sardinian RHS. The aim of this contribution is to stimulate a constructive discussion on the topic, to identify, finally, the necessary evidence-based health policies to be adopted.

## 2 Context—the current organization of the Sardinian regional health service

With the regional law no. 20 of September 11th, 2020, Sardinia dismantled the single health authority (ATS) established on January 1st^,^ 2017, pursuant to regional law no. 17 of July 27th, 2016. This political action reformed the regional health service (RHS) by reconstituting eight distinct local health authorities (ASLs); two university-hospitals (AOUs of Cagliari and Sassari); a highly specialized hospital named ARNAS-Brotzu; a regional emergency agency (AREUS); and a service’s agency (ARES). Presently, Sardinia counts 57 public hospitals and about 51 accredited private hospital facilities, whose activities should be subsidiary to the public health service.

Sardinia is one of the few Italian regions lacking a Scientific Institute for Research and Healthcare (I. R. C. C. S.) where translational research and clinical activities can be integrated, and highly specialized centers (hospitals with specific mission / vocation) where state-of-the-art treatments are provided. It also lacks a regional referral pediatric hospital.

The two University-Hospital Trusts in Sassari and Cagliari, which host the medical schools, along with ARNAS-Brotzu (a multi-specialty facility in the Cagliari area, with special focus in emergency care and transplants), are the metropolitan hospitals that handle the majority of patients, in particular almost all of those requiring medium-to-high complexity care.

Consequently, seeking healthcare outside the region for a wide range of medical and surgical specialties, particularly orthopedics, medical and surgical oncology, pediatric conditions, and neurological diseases, is a common practice among the Sardinian population (5). The island is also struggling significantly in the primary care sector, particularly in inland and mountainous areas where general practitioners are very few. Consequently, managing chronic conditions locally proves very difficult, and patients are inappropriately referred to the nearest hospitals, leading to overcrowding in emergency departments and inpatient wards.

With the regional law no. 8 of March 11th, 2025, the Government attempted to reform the system providing proposals for strengthening community medicine with the aim to reduce consistently the inappropriate access to the hospitals. The necessary and urgent definition of well-defined missions for each regional hospital has been also relaunched, as well as the need to establish a regional reference pediatric hospital (IRCCS) capable of combining the island’s first class genetic research with the childcare (6).

Despite the need of a functional reorganization of a regional hospital network, to rationally optimize the limited human and technological resources available, the law no. 8 was challenged by the National Government, which deemed article no. 15, concerning the proposed governance change of the health authorities, unconstitutional.

The Constitutional Court, with ruling no. 198, December 23rd, 2025, upheld the Government’s challenge on technically impeccable grounds. As a result, Sardinia has yet to define the governance of its local health authorities, which are still the subject of embarrassing political and party disputes.

## 3 Detail to understand key elements

### 3.1 Critical issues arising from the current organizational model of the Sardinian regional health service (RHS)

A RHS with a plethora of no-networked local hospitals, redundant and fragmented services and significant organizational challenges is definitively operative in Sardinia. This organizational model has dispersed throughout the region the already scarce clinical and administrative human resources available to date. Virtuous public hospitals with well-defined vocation, sufficient human resources and acceptable caseloads, which are fundamental for providing state-of-the-art healthcare to citizens, are very few in Sardinia and limited to the metropolitan hub facilities. This likely represents one of the most important criticalities for the RHS. Moreover, due to the reckless dispersal of expert administrative staff originally centralized in ATS, within the newly established multiple ASLs, the latter were unable to submit the financial statements for the years 2022–2024 to the National Government (Ministry of Finance) within the timeframe required by current regulations, resulting in management and planning chaos for at least 2 years.

A detailed survey of the public regional hospitals personally conducted on-site during the years 2024–2025 (in the capacity of Regional Councilor for Health and Social Policy) revealed that most of them have severe shortages of specialist medical doctors, nurses and administrative staff, resulting in organizational challenges and failure to provide adequate health services. Several surgical departments have been inexplicably created in peripheral hospitals without sufficient specialist teams and technologies available; consequently, medium- to high-complexity surgical procedures that require a multi-specialist approach cannot be guaranteed in these contexts.

The scenario is further complicated during the summer season (June–September), when the Sardinian population triples in size, as the island is a highly attractive tourist destination.

Emergency departments and intensive care units, where they exist, face truly critical situations.

As expected, almost all patients referred to local hospitals are diverted to the metropolitan hospitals of Cagliari and Sassari, the only ones capable of managing medium-high complexity cases, even though their emergency departments and clinical wards are frequently overloading, with occupancy of beds exceeding 150% during most of the year (7).

### 3.2 Insularity, orographic characteristics and scientific isolation of the RHS and their potential impact on the health services performance

Insularity and transportation difficulties, both internally and to the mainland, represent two of the main critical issues facing the Sardinia Region. Much of the population living in the inland areas has difficult access to healthcare services provided by the main metropolitan hospitals.

Likely, this is one of the reasons why many regional peripheral hospitals have been realized, serving primarily as municipal hubs for emergency access and low-to-medium intensity care.

Unfortunately, in contexts like these, where limited and dispersed human resources and technological skills are invariably registered, it is almost impossible to provide diagnostic-therapeutic care pathways (DTCP) at the state-of-the-art, for the most relevant pathologic conditions. As a subtle consequence, not fully understood by the local and regional politicians, a slow and inexorable loss of specialist medical doctors is registered in the region. In an entropic context where all the hospitals provide the same “basic health services” and where “everyone does everything,” often outside the national guidelines, it will be very difficult to provide the minimum levels of qualified and state-of-art healthcare services, according to the good clinical practice, and most important, in complete safety (8). In such contexts it is almost impossible to attract and then retain specialized medical doctors, create schools and achieve significant qualified caseloads.

As a result, the quality of healthcare services in the regional local hospitals is going to inexorably decline. It is therefore not surprising that 16% of physicians at the University Medical Schools of Sassari and Cagliari leave their jobs within 3 years of obtaining the specialist degree, opting for more challenging and professionally rewarding positions in other Italian Regions or even abroad.

This critical situation is compounded by the inevitable burnout of healthcare workers at the main metropolitan hospitals, particularly in the emergency departments, due to workloads that have become unsustainable for the continuous transfers of patients from the inland areas.

Altogether, lack of an efficient regional hospital network, the shortage of medical and nursing staff in unattractive outlying hospitals, and internal transport difficulties, have turned a chronic emergency into an explosive situation.

The condition of insularity is partly responsible for other critical issues.

The limited capacity of collaboration and dialog with leading Institutions on the mainland, is more than evident and further compromised by the environmental and cultural isolation.

The Businco Oncology Hospital of Cagliari, for example, is the only Sardinian hospital with a well-defined oncology mission. Unfortunately, its clinical and surgical activities need to be consistently strengthened. This “specialized hospital” is not part of the National Oncology Hospital Network worded “Alliance against Cancer” founded in 2002 by the Italian Ministry of Health (9) which aims to translate research innovation into clinical practice and standardize cancer care across the country, particularly regarding equitable access to innovative drugs (10).

For this reason, participation of Sardinian patients to clinical trials at national and international level is very difficult to date. Clinical and experimental trials that use molecular targeted therapies and innovative therapies coordinated by local oncologists, if any, are not registered. Only recently with the Regional resolution no. 42/61 of November 6th, 2024, Sardinia has attempted to improve access to innovative drugs, which did not exceed 4% compared to the national average of 50% (7, 10, 11). Surgical oncology at Businco Hospital is mostly restricted to breast and lung cancers with procedures that were not always fully compliant with the national guidelines until the years 2023–2024. The volume of cases treated for other types of cancer is very low and below safety thresholds.

In the AgeNaS PON 2025 no state-of-the-art facilities for oncological surgery have been identified in Sardinia (Figure 1). Very recently the Regional Government finally recognized the urgency to create a Regional Oncology Network interfaced with the major National Cancer Institutes in the mainland. The aim was to align the oncology activities on the island to the national standards, strengthening both clinical activities and translational research, improving the access of Sardinian patients to clinical trials, which use innovative therapies (7, 10).

Until the end of 2025, Sardinia, as autonomous region, was barred from accessing the National Fund for Innovative Drugs provided by the Italian Medicines Agency (A. I. F. A.) for ordinary statute regions. Recently the National Government has changed the rules and starting from 2025 Sardinia has been finally granted (11).

This represents a concrete opportunity for Sardinia to introduce molecular therapies in many clinical areas, particularly in oncology, when DTCP for each tumor type will be created and networked hospitals with specific oncology vocation finally defined and empowered. This functional organization will likely reduce patients’ distress and costs for extra-regional healthcare mobility as well.

### 3.3 Genetics, character traits and relational peculiarities of the Sardinian people

Sardinian population has a unique genetic heritage, strongly influenced by insularity. Relevant genetic studies have demonstrated a high frequency of genetic variants in Sardinians, associated with traits such as resistance to malaria and adaptation to the environment (12–17).

Particularly noteworthy is the high concentration of healthy centenarians in certain areas of the island, referred as blue zones, and the highest incidence of some rare genetic diseases, compared to the rest of Italy and Europe (1, 18). These data demonstrate the existence of genetic peculiarities that make Sardinia unique in many ways.

In addition to influencing the prevalence of certain diseases and contributing to environmental adaptation, genetics determines also the predisposition to certain personality traits, such as touchiness, aggressiveness, introversion, divergent thinking (19).

Gene expression is certainly influenced by epigenetic environmental factors such as diet or lifestyle, but the character trait of a population is the result of complex interactions between genetic, environmental, cultural, and historical factors that all together contribute to shaping it (20).

*Pocos, locos e male unidos* is a Spanish expression meaning “few, crazy, and poorly united,” used by the Spanish King Philip II way back in 1581 to describe the Sardinian population, considered rebellious, scattered across the country, and difficult to govern (21).

The expression emphasizes the perception of a certain independence and resistance of Sardinians to foreign rulers. This fact, deeply rooted in the oral and written tradition of Sardinian people

should not be considered *stricto sensu* as “racial reticence “but rather a historical response to centuries of colonialism.

The theme is a recurring one in non-fiction, anthropology, and Sardinian literature from the 19th century to the present day. English speakers define it as “Sardinian reserve,” “island mentality,” “code of silence,” “colonial reticence” (21–27).

In the Martha King’s widely cited translation of the novel “Reeds in the Wind” by the Sardinian writer and Nobel laureate Grazia Deledda, the author aptly describes the character trait of “Sardinian reticence” and explains its origins. The concept is vividly portrayed: ….*” the people of Sardinian are marked by a centuries-old reserve, a silence born of repeated foreign domination*….(omitted) …*To survive the Sardinian learned not to speak, not to reveal himself to the outsider”* (23, 24, 27). In “The Forest of Norbio” written by Dessì, the protagonist, coming from the mainland, never truly manages to enter the island. The Sardinians therefore “remain shadows” (25). Various historical and anthropological contributions on the subject are well-documented in the English-language literature as well (22, 26).

But what does this have to do with regional healthcare?

Distrust and closure to the external comparison is a distinctive character trait deeply rooted in this regional context and is quite evident even today.

Although art and literature are relatively immune to this environmental and cultural isolation (Sardinia has been able to produce eminent writers, sculptors and musicians), complex scientific fields such as basic sciences, medicine, bioengineering and so on, remain inexorably conditioned by such a peculiar character trait. Research and development in medicine and biomedical sciences invariably require an open mind and ongoing dialog with the scientific community at National and International level. This improves fundamental knowledge that will allow prompt access to new diagnostic and therapeutic technologies and more in general to innovations. Mistrust, a refusal to engage with others, a reluctance to showcase one’s expertise and the results produced in a critical context, and self-referentiality contribute all to the system’s progressive isolation and backwardness in various scientific fields, medicine foremost.

As discussed above, it is pacific that the progressive decline of the Sardinian public health system, one of the richest in Italy considering the small number of inhabitants, cannot be attributable to the lack of economic resources. Moreover, the health policies implemented for a couple of decades by consecutive Regional Governments of different political persuasion did not reverse the negative trend, suggesting that a common lack of technical-political vision and/or local political interferences could be critical determinants of the RHS’s failure. An alternative possibility is that awareness of the technical complexity of the work necessary to improve the performance of the RHS could be the hidden excuse for political immobility (28).

### 3.4 Policy and local balances

**Figure 3:**
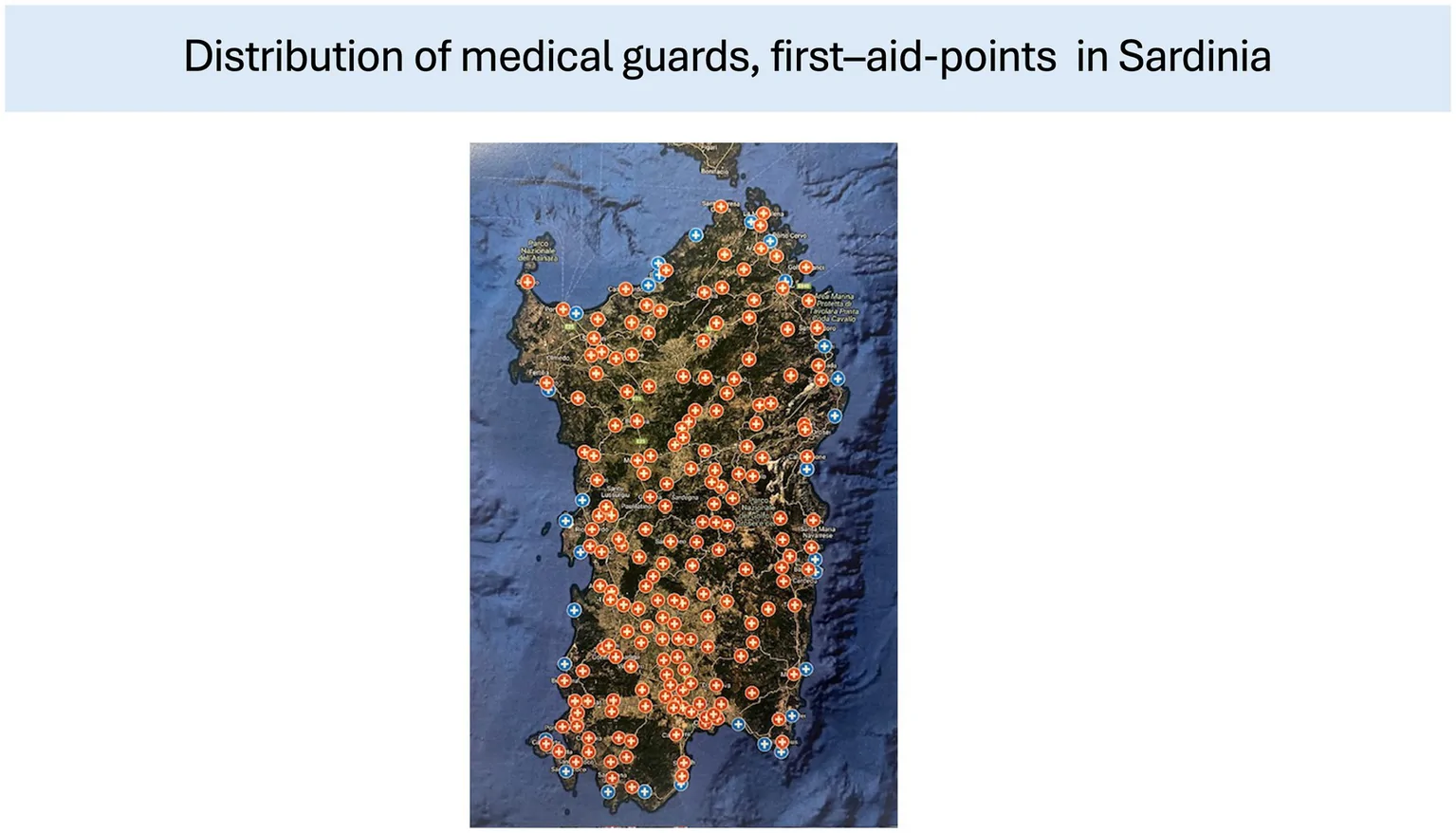
Distribution of redundant and non-functional medical guards and first-aid-points in Sardinia (data from AgeNaS, the National Agency for Health Services and Regional Health Department—ASLs, Sardinia; in blue are those first-aid-point activated along the coast during summer).

The peculiar character of Sardinian people and the sociocultural context, have transformed the necessary and urgent cross-cutting actions of health policy into approximate and fragmented political ads, primarily aimed at obtaining local consensus rather than safeguarding the citizens’ health. A paradigmatic example is represented by the plethora of first aid stations (193 compared to 92 needed as estimated by AgeNaS) accomplished by local politicians in every Sardinian municipality to catch electoral consensus, despite the awareness of the serious shortage of healthcare personnel for ensuring their effective function (Figure 3). It is not surprising, indeed, that over 60% of these structures remain empty and not operative.

In Sardinia, almost all politicians elected in the Regional Council deal with healthcare, a politically strategic topic for the target audience and the financial resources manageable. Unfortunately, only 4 out of 60 Councilors in the current Regional Government are medical doctors with specific technical competences. Moreover, and worthy of reflection, the organization of the Regional Department of Health and Social Policies appears paradoxical: about 180 employees, two General Directorates, four Service Directorates, one cabinet staff, but no medical doctors except one part-time consultant. Even pharmacologists, biologists and epidemiologists are absent or isolated figures. It is pacific that in this context is almost impossible to define the necessary evidence-based actions required to definitively improve at structural and functional level the RHS.

The nature and quality of the healthcare policies implemented over the years can be easily gleaned from the deliberative activities of the Regional Executive Board (7). High-quality, cross-sectoral policies based on rigorous technical and scientific analysis, potentially capable of impacting RHS efficiency, are missing. Moreover, the amendments proposed by Regional Councilors during the approval of financial laws are generally uncoordinated and fragmented initiatives, mainly aimed at promoting the political visibility of the council groups in the respective territories, but very far from the goal of addressing urgent structural problems in the regional healthcare system.

A couple of examples may clarify this critical point:

- Up to 10 million euro have been recently allocated for the so-called “Health Vouchers” to allow indigent patients to undergo gastrointestinal endoscopic procedures and other health services, strictly provided at private healthcare facilities on the territory, at the expense of the Region;
- 600.000 euros have been allocated to purchase generic hyperbaric therapies (for which there is not a critical waiting list) provided by private facilities also.
- A regional allowance for fibromyalgia of €800 *una tantum*, was created to reimburse undefined needs not covered by the RHS. In the year 2023, €5.1 million were allocated to satisfy 6,396 applications. Less than 1 year later, up to €7.5 million were necessary to cover 9,392 applications. The sudden increase of fibromyalgia cases in less than 1 year, would have merited a focused epidemiological study, which was unfortunately not feasible because “politically inappropriate.” These and dozens of similar initiatives, very clever from the electoral point of view to gain political consensus, have unfortunately overshadowed for the umpteenth time, the real health needs: for example, to strengthen cancer prevention initiatives, which are woefully inadequate in the region [in the year 2026 the regional tumor registry is still missing!; (2, 7)].

The analysis of data collected in the field over 20 months of work (April 2024–December 2025) clearly demonstrates the critical state of the RHS; urgent and unpostponable structural and functional changes are necessary to finally reverse its decline. The professional isolation of healthcare personnel is also clear, as well as the embarrassing cultural distance of a political class technically unprepared to adequately respond to the cries for help from both Sardinian doctors and citizens, yet highly skilled at building consensus around itself.

Among the most heated political battles in Sardinia, is the appointment of the General Directors of the Local Health Authorities (ASLs), positions that have always been the source of fierce political conflict between Sardinian parties. It seems more than evident that a Sardinian-born General Director of a local health authority, chosen by the dominant political fraction, is much more suited to gaining local electoral consensus than a competent external expert capable of rationally resolving the structural and functional problems of the health care facilities he is to manage.

For decades, the general directors of the Local Health Authorities (ASLs) have almost all been Sardinian, some of whom undoubtedly competent, but invariably chosen through exhausting negotiations between the Regional Government and the most influent local political fractions, irrespective of any technical-scientific expertise. This ensures the hermetic closure of a complex relational system like healthcare, protecting it from the risks of a politically unmanageable renewal, especially if proposed by “foreign rulers.”

This *modus operandi*, completely disregarding the true needs of public health, is still in use today, and its negative impact on the RHS’s performance is visible and measurable.

Patients’ associations in Sardinia are also politically very active. It is easier to allocate 7 million euro to support patients with fibromyalgia, with a freely spendable, non-accountable health bonus, than renovating the embarrassing and non-compliant surgical operatory rooms of the unique oncology hospital of the Region, for which restricted funds were already allocated by the European National Recovery and Resilience Plan (NRRP). The fierce protest that took place in 2025 by some cancer patients’ associations demanding the halt of the works was astonishing!

Probably, modernization of the operatory rooms of the oncology hospital fails to generate immediate political consensus, despite it will resolve several structural, logistical and clinical problems for cancer patients that cannot longer be postponed.

Allocation of resources for private healthcare facilities, for provision of diagnostic tests and other healthcare services on behalf of the RHS, represents another important and particularly costly initiative implemented by the Region yearly, with the specific aim of reducing the waiting lists for EPC. To date, the spending caps for accredited private laboratories, diagnostic facilities and private hospitals have been clearly defined, but the type and number of healthcare services they should provide to the population have never been precisely indicated (7).

As a corollary, it seems clear that the direct management of local health policies is of cross-cutting interest, as it is highly profitable in terms of political consensus.

This scenario could likely explain the long-standing inertia of all successive Regional Governments, whose policies invariably failed to bring any objective improvement to the RHS.

It is evident by now, that healthcare governance detached from local political interests has been highly unwelcome in Sardinia for many years, especially in the presence of “foreign rulers” potentially able to provide the required trimming of the system. An evidence-based approach does not reward the politic of consensus, but it could conceivably be beneficial for revitalization of the RHS.

## 4 The proposed functional reform of the RHS (regional law no. 8 of march 11th, 2025)

The need for a comprehensive structural and functional reform of the Sardinian RHS appears even more necessary when we examine, from a strictly technical perspective, the major critical issues that have arisen over the last 20 years and are leading to its progressive decline (Table 2).

**Table 2:** Major critical issues impacting the Sardinian’s RHS.

Critical issues in Community Medicine Shortage of general practitioners and pediatricians Poor efficiency in taking charge of chronic conditions Lack of prevention
Critical issues in Regional Local Hospitals Redundant and poor-quality healthcare services. Lack of a regional hospital network. Lack of hospitals with well-defined mission/vocation. Lack of hub-&-spoke organization for comprehensive care. Lack of core facilities for diagnosis and therapy of complex diseases Caseloads dispersion; hospital facilities not attractive for specialized medical doctors Operational criticalities for major surgery and emergency care Long waiting lists.
Critical issues in University Hospitals (AOU) and Metropolitan Hospitals Critical graduated medical schools for the RHS, lost. Lack of a Regional referral hospital for pediatrics and rare diseases (IRCCS) Limited translational research in biomedical sciences and Medicine.
Critical issues in Health Policies Scarce or absent cross-cutting and evidence-based health policies adopted. Allocated funding for visionless, non-functional health initiatives for the RHS Local and regional health policies mostly adopted to gain electoral consensus. Lack of medical doctors, biologists, pharmacologists and epidemiologists within the Regional Health Department, at structural level.

A recent proposal for a functional reform of the RHS has been presented to the Regional Council with the law no. 8 of March 11^th^, 2025 (6).

The three most significant actions proposed were:

1. *Reorganizing community medicine according to demographics and healthcare needs of the Sardinian population.* In addition to renewing the contractual agreements with general practitioners and pediatricians, to manage chronic conditions outside the hospital, the establishment of three Regional Reference Centers (RCs) has been proposed, aimed to achieve maximum proximity care on each regional territory: (i) the RC for mental health and addictions, (ii) the RC for rehabilitation, and (iii) the RC for prevention. Each RC plays as coordinator hub for homologous departments (spoke) present in the territories. This strategy should overcome consistently the hospital-centric vision of healthcare, limiting the direct access to the hospitals to patients with acute conditions and complex emergencies. The model appears coherent with the necessity to strengthen the community health network at National level, with creation of the so-called Health Houses envisaged by the National Recovery and Resilience Plan (NRRP), founded by Next Generation EU with 2 billion euros.
2. *Creation of hub-and-spoke hospital networks, enabling hospitals with similar vocations to provide state-of-the-art healthcare services, exploiting the existing resources available in house and enhancing them where necessary.*

This initiative optimizes patient care and quality of treatment provided, by promoting a coherent and optimal redistribution of human resources and technology across a few, well-organized hospital facilities. The goal is to ensure comprehensive care for patients with specific pathologic conditions, from the diagnostic process to follow-up. This organization ensures full compliance with the Diagnostic-Therapeutic Care Pathways (DTCP) guidelines.

For example, after a thorough review of the available skills and technologies on-site, three hub Centers for breast surgery were identified in the South of the Region (Cagliari), in the Center-North (Nuoro), and in the North-West (Sassari). Sardinian patients with suspicious breast cancers can be directly referred to these facilities and promptly included in the diagnostic-therapeutic pathways in appropriate timeframe (7). Diagnosis and surgical removal of the tumor with simultaneous breast reconstruction are finally executable in Sardinia, as well as the phenotypic and molecular tumor profiling that allow tailored oncological therapies. These procedures were not invariably guaranteed until the end of 2024.

This functional re-organization may be applicable to other pathologic conditions to allow the provision of state-of-the-art healthcare services, while optimizing technical and human resources and minimizing the costs of extra-regional healthcare mobility.

1. *To* r*eorganize the governance of the Local Health Authorities (ASLs) rationally, based on a rigorous, objective technical vision, which must not be influenced by local political interests.*

This represents the most critical point of the reform, which is more difficult to implement in this specific regional context. In other words, it means “decouple local and regional political influence from the technical corrections urgently required for the proper functioning of the RHS.” To date, despite the five-year alternation of Regional Governments with opposing political visions, this objective is far to be achieved.

## 5 Conclusion and future perspectives

To reverse RHS’s negative trend a substantial cultural shift is needed in the relationship between technical and political spheres, which should be increasingly integrated and not antithetical.

At stake are the coherent development and sustainability of the RHS, which must dynamically engage with technical and scientific progress. The integration of technical skills at political level is imperative and cannot be further ignored, to guarantee the rapid access to innovation and state-of-the-art healthcare services for Sardinian people.

The Sardinian healthcare system is currently a decade behind the other Italians Regions, due to cultural isolation, self-centeredness, reluctance to bring in skilled medical doctors from the Continent to cover apical position and create schools, and most importantly, for a political system that has long proven to be skilled at gaining instant consensus rather than structurally solving the citizens’ health problems.

The most important healthcare policies that urgently need to be adopted should include:

(a) actions to improve the community medicine for optimal local care of chronic patients; (b) to implement new technologies such as artificial intelligence and telemedicine to reduce waiting lists and unnecessary admissions to hospitals; (c) to promote a virtuous subsidiary integration between the RHS and accredited private healthcare facilities to facilitate access to services and reduce waiting lists; (d) to invest in prevention, in gender medicine and in the “one health approach to healthcare.

At the same time, specific structural actions are necessary to improve the efficiency of regional hospitals: (e) defining the mission/vocation of each hospital by implementing specific expertise.

(f) Creating a regional hospital network according to the hub-and-spoke model by pathology, concentrating caseloads and overcoming the geographical dispersion of human and technological resources. (g) To promote skills and to invest in research and development should be the priority mission of the university hospitals (AOU), adequately supported by the Regional Government.

Concluding, although some of the critical issues outlined may be considered common to all Italian Regions, the condition of insularity highlights the most relevant ones, without distorting effects. Field observation of the problems affecting the Sardinian RHS strongly suggests that it is time to prioritize its structural and functional reorganization by using a rigorous evidence-based approach. This objective does not yet seem within the reach of a disoriented and quarrelsome political class that, on the one hand, could use the complexity as an alibi for inconclusiveness, but on the other side does not yet understand that the good health policies aimed at protecting public health, invariably require high-level technical expertise, evidence-based decisions, and cross-cutting political consensus.

## Data Availability

The raw data supporting the conclusions of this article will be made available by the authors, without undue reservation.
